# Heat Transfer Behavior across the Dentino-Enamel Junction in the Human Tooth

**DOI:** 10.1371/journal.pone.0158233

**Published:** 2016-09-23

**Authors:** Lin Niu, Shao-Jie Dong, Ting-Ting Kong, Rong Wang, Rui Zou, Qi-Da Liu

**Affiliations:** 1 Key Laboratory of Shaanxi Province for Craniofacial Precision Medicine Research, College of Stomatology, Xi’an Jiaotong University, Xi'an, Shaanxi, China; 2 Clinical Research Center of Shaanxi Province for Dental and Maxillofacial Diseases, College of Stomatology, Xi’an Jiaotong University, Xi'an, Shaanxi, China; 3 Stomatology Hospital of Xi’an Jiaotong University College of Medicine, Xi'an, Shaanxi, China; 4 State Key Laboratory for Strength and Vibration of Mechanical Structures, Xi'an Jiaotong University, Xi'an, Shaanxi, China; Georgia Regents University, UNITED STATES

## Abstract

During eating, the teeth usually endure the sharply temperature changes because of different foods. It is of importance to investigate the heat transfer and heat dissipation behavior of the dentino–enamel junction (DEJ) of human tooth since dentine and enamel have different thermophysical properties. The spatial and temporal temperature distributions on the enamel, dentine, and pulpal chamber of both the human tooth and its discontinuous boundaries, were measured using infrared thermography using a stepped temperature increase on the outer boundary of enamel crowns. The thermal diffusivities for enamel and dentine were deduced from the time dependent temperature change at the enamel and dentine layers. The thermal conductivities for enamel and dentine were calculated to be 0.81 Wm^-1^K^-1^ and 0.48 Wm^-1^K^-1^ respectively. The observed temperature discontinuities across the interfaces between enamel, dentine and pulp-chamber layers were due to the difference of thermal conductivities at interfaces rather than to the phase transformation. The temperature gradient distributes continuously across the enamel and dentine layers and their junction below a temperature of 42°C, whilst a negative thermal resistance is observed at interfaces above 42°C. These results suggest that the microstructure of the dentin-enamel junction (DEJ) junction play an important role in tooth heat transfer and protects the pulp from heat damage.

## Introduction

Thermophysical properties of the human tooth, such as thermal diffusivity and conductivity impact heat transfer and thus are of great significance in the design of dental filling materials and equipment [1–3]. These thermophysical properties are also important in the pathophysiology of thermally induced pain and damage [4–7]. Thus, the restoration of a tooth may involve decisions about materials such as ceramic systems or cements [8,9]. Knowledge about the heat diffusivity of different materials is potentially important for clinical decision making in addition to the usual considerations concerning tooth preparation, cement system and disease pathology [10–12]. Obviously, a simple yet reliable experimental measurement for determining thermophysical properties is needed.

Mature enamel is composed almost entirely of mineral (>95% per volume) bundled with highly elongated crystals while dentine consists of 50vol% crystals, 20vol% water, and 30vol% organic matrix. From a materials’ point of view, a human tooth is composed of a peripheral enamel layer of high material strength and hardness bonded to a softer but tougher substratum (dentine) with the dentino-enamel junction (DEJ) in between. The DEJ is a 100~150μm broad transitional zone region at the material interface [13–15]. Its mechanical function is to bear loading during mastication and thus the DEJ plays a key role in resisting crack propagation and to protect the enamel layer from delamination from the dentine core [16,17]. Bai et al. [13] reported that microcracks initiate at the DEJ and spread into the enamel side due to the differences in the hardness/elastic modulus between dissimilar materials. There is a large amount of data on the mechanical behavior of the DEJ but relatively little on the thermal behavior of the DEJ. This is due in part to variations in the material composition of DEJ, complicating this calculation [15,18,19]. However, measurement of the thermal capacity (cp) is possible with either differential scanning calorimetry (DSC) or differential thermal analysis (DTA).

Teeth generally have poor heat transfer properties and their overall thermal conductivity is about 0.6 Wm^-1^K^-1^[20,21]. This creates methodological measurement challenges in addition to the numerous functional, biological, and geometrical structure complexities [22,23]. To address these technical challenges, we used infrared thermography for the heat transfer studies because it provides a non-invasive method for measuring spatially resolved surface temperature distributions, even when large surface temperature gradients are present [24]. A widely used method for the study of heat transfer behavior of the dentino-enamel junction involves use of thermocouples but is incapable of measuring the surface temperature distribution of interest. In contrast, infrared thermography provides a one step measurement of a number of other relevant parameters including surface heat transfer coefficient distributions, non-dimensional quantities containing surface heat transfer coefficients, surface adiabatic effectiveness and thermal boundary conditions. Thus, this non-invasive method for temperature measurement (infrared thermography) has been validated as being capable of measuring temperature variations in small biological tissues with high accuracy.

By convention, the thermal diffusivity for a tooth measured using time dependent changes in tooth temperature is called the monotonic heating regime method. In the experiments presented below we extend this method with IR-thermography to measure time-dependent changes in temperatures at the dentino-enamel junction. The data obtained by this method can be used to determine the thermal conductivity of dentine and enamel of human teeth and for studying the heat transfer behavior at their junction.

## Materials and Methods

### Ethics Statement

All the experimental procedures involving human tooth were approved by the Ethics Committee of Stomatology Hospital of Xi'an Jiaotong University College of Medicine (Permit Number: 2015524). All patients provided written informed consent.

### Sample preparation

Although individual teeth have wide variations in surface areas, for the same kind of tooth the ratio between the surface area of the enamel and that of the dentino-enamel junction are very similar. The area of the enamel, dentine and the dentino-enamel junction actually encompass a three-dimensional structure that makes it difficult to study heat transfer behavior. Six human mandible third molars from six different patients were used in the present study, a representative sample of sectioned tooth that included the dentino-enamel junction was used and calculations were based on a simplified model of heat transfer between the enamel surface and the underlying tissue. Heat transfer measurements were based on the thermal-physical differences between dentine, enamel and the DEJ.

**Fig 1:**
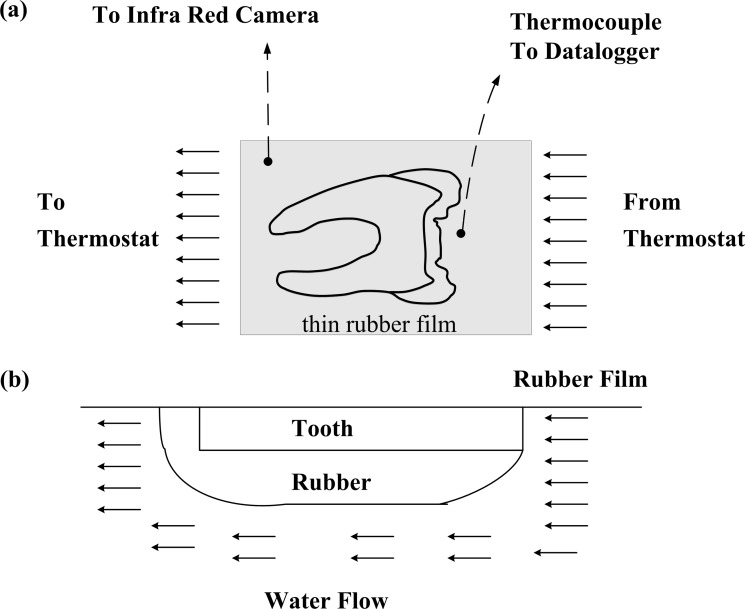
Diagram of steam chamber with sample and water steam. (a) top view (b) side view. Note that the specimen was machined from a human 3rd molar sliced in the buccal direction and with the in-plane tubules perpendicular to heat flow direction.

The samples used in this study were from a section of an adult mandibular third molar, which was collected from patients undergoing routine orthodontic extraction at the University Dental School. The extracted tooth had its periodontal tissue removed and was sterilized by 75% alcohol prior to storage at 4°C in PBS buffer solution. The sample was about 2.5mm in thickness cut by a diamond blade microtome (Buchler Ltd. ISOMET 1000); the cutting surfaces were parallel to the buccal and perpendicular to the lingual surfaces and one surface polished to a 3-μm-particle-size paste (Buchler Ltd.). The sample had characteristics of an enamel boundary and varied with the thickness of the enamel layer (Fig 1A).

Subsequently, it was embedded into the 0.5×150× 150mm container with a thin layer of a mixture containing a resin powder with the curing agent; a flat sheet of acrylic is formed by a natural drying process after about 48 hours. Note that this results in a solid resin plate with the sliced tooth in the center. The acrylic was chosen because of its excellent performance in thermal insulation (the thermal conductivity was about 0.16 Wm^-1^K^-1^ at 20°C) and is easily handled; it was further used to apply the thermal insulation boundary for the given sample surface by applying a thick layer of the material on the sample.

### Experimental arrangement

Schematic diagram of the setup employed for heat flux reduction measurements using infrared imaging as Fig 1 shows [24]. The plate with the sliced tooth sample was fixed to the poly-tetrafluoroethylene (PTFE) stand chamber and a thin rubber film was mounted on the top to prevent water leakage. The lingual surface of the sample was water heated and the water temperature was controlled by a thermostat on the water tank (Fig 1). Surface temperature data were collected using an infrared camera (NEC, TH9100 MV) mounted perpendicularly to the direction of the water flow, and pointed towards the DEJ. A window made of sodium chloride was used to allow maximum transmission of radiation emitted from the surface. The camera was calibrated by comparing camera-detected temperatures to values measured with J-type thermocouples mounted at several places close to the lingual surface; calibration details are as previously described [24]. The variability of the surface temperature measurements was determined based on the curve fit error for the linear calibration equation, which was about ±0.2°C. The spatial resolution of the infrared measured surface temperatures was about 10 times the thickness, where discrete pixel levels of 240×320 pixels are based on integrated averages over a 24×32 mm^2^ surface area. This resolution was adequate to resolve measured temperature gradients of the DEJ.

The volume of water in the thermostat was about three orders larger than that of the PTFE stand chamber, and thus the temperature of water in tank was quite stable (±0.1°C). Water circulation in the stand chamber to the water tank was driven by the pump located in the water tank. Before starting the water circulation, the PTFE stand chamber was filled with water at room temperature (22.5°C) and then the water was heated to 60°C. Replacing the water in the PTFE stand chamber takes approximately 12 seconds.

### Infrared imaging and data acquisition procedures

Spatially resolved temperature distributions along the sliced tooth surface were determined using infrared imaging in conjunction with thermocouples, digital image processing, and *in situ* calibration procedures. To accomplish this, the infrared radiation emitted by the heated polished tooth surface was captured using a NEC, TH9100 MV infrared imaging camera, which operates at infrared wavelengths from 8μm to 14μm. The tooth surface, as it was tested, was mounted on a flat sheet of acrylic. Temperatures were measured using calibrated, miniature copper–constantan thermocouples (0.5mm in diameter) distributed along the tooth surface being measured and adjacent to still air, with an aim to perform the *in situ* calibrations simultaneously as the radiation contours from surface temperature variations were recorded.

Three thermocouple junction locations were present in the infrared field captured by the camera. The exact spatial locations and pixel locations of these thermocouple junctions and the coordinates of the field of view were known from calibration. During this procedure, the camera was focused, and rigidly mounted and oriented relative to the test surface in the same way as when radiation contours were recorded. Voltages from the thermocouples were acquired using an Agilent 34970A data-acquisition system, controlled by a PC computer. Images from the infrared camera were also recorded as 8-bit gray scale by the PC using a frame-grabber video card and associated software. It was assumed that the microscopic level crystals had a uniform width and thickness and that the angle of emergence of the crystals observed was sufficient to provide the required increase in the enamel surface area.

## Results

### Transient surface temperature distribution

**Fig 2:**
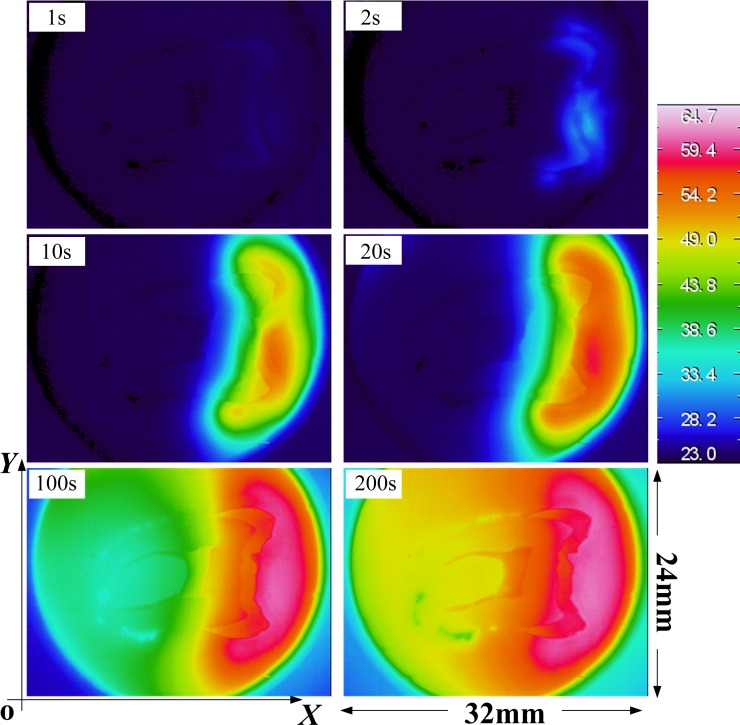
Images acquired 1, 2, 10, 20, 100 and 200 second from the beginning of testing of a tooth section surface acquired to determine spatial and temporal surface temperature variations. Each image in two directions corresponds to an area of 32mm (x) by 24mm (y).

Fig 2 shows a series of infrared images acquired at 1s, 2s, 10s, 20s, 100s and 200s from the start. Here, x and y represent the coordinates in two directions along the sliced tooth surface shown in Fig 2. Infrared camera images are shown in the left-hand columns, with associated surface temperature contour bar presented in the right-hand column. Notice that the highest temperatures are located near the center of the enamel surface for the 20s data sets. As time proceeds, the shape of the dentino-enamel junction becomes clear and temperature levels increase from the right to the left; this is because the left portions of the patch heat up as energy is conducted away from the right patch (water). The time variations of the maximum temperature and spatially averaged patch temperature are shown in Fig 2.

Shown in Fig 2 are the infrared camera images of spatially resolved temperature distributions. Increasing temperatures are shown from right to left, applied by a convective heat transfer process at the interface between the crown and water flow. Although the crown surface in contact with the water flow is a three dimensional structure and the heat transfer is a dynamic process (time-dependent), this was not expected to significantly affect the current results. Since only the crown is heated and the other surfaces are insulated, the temperature should rise gradually from the initial state to the steady state. This kind of boundary condition gives a uniform temperature distribution on the tooth surface at the initial state and steady state as be seen in the 1s and 200s images.

**Fig 3:**
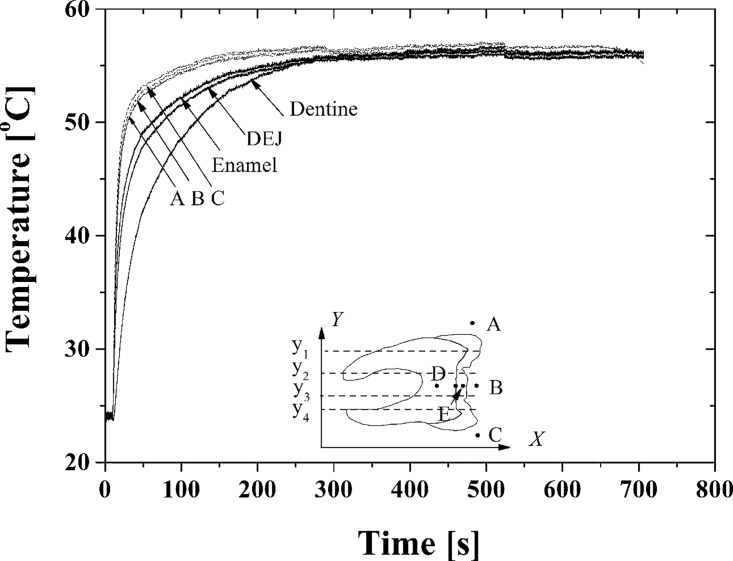
Time dependent variations of in at specific locations from tests on the sliced tooth surface, where the positions of A, B and C correspond to three positions in the water region and (D, E) are the dentine and enamel material region.

In Fig 3 temperature data is highlighted at points A, B, C, D and E. Points A, B and C correspond to points in the water region used to check the temperature loading by water circulation; three curves are collapsed into one which suggests that the water circulation system works effectively; the temperature increase at the initial point of the curve is rapid, similar to step loading. Higher temperatures could be utilized by increasing the water tank temperature but this was not done because it was not felt to be clinically relevant. The origin of the reference coordinates for the tooth specimen takes the pixel at the lower left corner of the images shown in Fig 2. The temperature at any given point can be calculated from the image sequences. We next examined, the “dentine”, “enamel” and “DEJ” locations, in relation to the water temperature regions. Fig 3 shows the temperature at these positions, where the “Dentine” and “Enamel” are denoted by “D” and “E”, while the “DEJ” is the natural boundary between them. Due to the different thermal properties of dentine and enamel, the temperature at “DEJ” is clearly distinguishable in Fig 2 and is plotted vs. time in Fig 3.

As seen in Fig 3 the temperatures at A, B, C, Dentine, DEJ, and Enamel are quite different within 300s. The temperature measurements at A, B and C using the IR-thermography were confirmed by the water immersed thermocouple. The measurement results at these three points are consistent well with each other within 300s; it suggests the validity of the chamber design and the efficiency of the heat convection system. As expected, the water temperature was much higher than that of dentine, DEJ or enamel within 100s but the differences became smaller after 300s. The data for Fig 3 represents a logarithmic type of curve since the temperature boundary state at the outer surface of crown is close to the Heaviside function (see the A, B, and C curves). Each curve provides the transition time or material relaxation time (*τ*), which relates to the thermal diffusivity (α). For calculating *τ*, we selected the central point of each material region at each layer as previously described [25] and the resulting calculated the *τ* for enamel (*τ*_*E*_), DEJ (*τ*_*J*_) and dentine *τ*_*D*_ region were 67.0s, 69.5s and 82.2s respectively. It is important to note that the order is *τ*_*D*_ > *τ*_*J*_ > *τ*_*E*_ and *τ*_*J*_ is close to the value of *τ*_*E*_.

Each pixel corresponds to the tooth surface of 0.1mm by 0.1mm and DEJ thickness is also about 100~150μm [15]. Therefore, the DEJ location can be determined by using the discontinuous temperature distribution across the junction. On the other hand, the measured width of tooth surface is about 2 orders of the spatial resolution of a pixel (<100 μm); and thus the transient heat conduction along each buccal line approximates to the solution of the temperature in a one-dimensional composite slab. In this model, the explicit expression of multi-layer transient heat conduction problems is exploited by analyzing the different transition times of the various layers of a composite slab as detailed below.

### Determination of thermal properties for dentine and enamel layers

The thermal properties of human teeth depend on material-dependent responses including increases in temperature, phase transition, volume change, or change of some other physical quantity. In this section, the physical behavior of the dental structure was studied using the degree of isotropy at a temperature of 60°C, which is applied to minimize collagen denaturation. It is possible to model multilayer transient heat conduction problems as various layers of composite slab geometry as is shown in Fig 1A. The eigenvalues of the composite slab are analyzed and estimated using the transient temperature data measured at two points along the direction of heat flux on the material plate as shown in Fig 1B, *α*_1_ is approximately determined by the enamel layer[24,26–29],

$$\alpha_{1}=\frac{4}{\pi^{2}}\frac{L_{1}{}^{2}}{\tau_{2}-\tau_{1}}.$$ (1)

and within the dentine layer *α*_2_ is calculated by

$$\tau_{c}=\frac{\tau_{2}-\tau_{1}}{\tau_{3}-\tau_{2}}=\frac{\gamma^{2}}{\delta }=\frac{{\left(L_{2}/L_{1}\right)}^{2}}{\alpha_{2}/\alpha_{1}}.$$ (2)

Using the values of *L*_2_ = 2.5mm and *L*_1_ = 1.6mm, and the measured thermal diffusivities *α* of the enamel and dentine materials are 4.2×10^-7^m^2^ s^-1^ and 2.6 ×10^-7^m^2^ s^-1^, corresponding to the thermal conductivities of enamel and dentine are 0.81 Wm^-1^K^-1^ and 0.48 Wm^-1^K^-1^.

### Negative thermal resistance at the dentino-enamel junction

**Fig 4:**
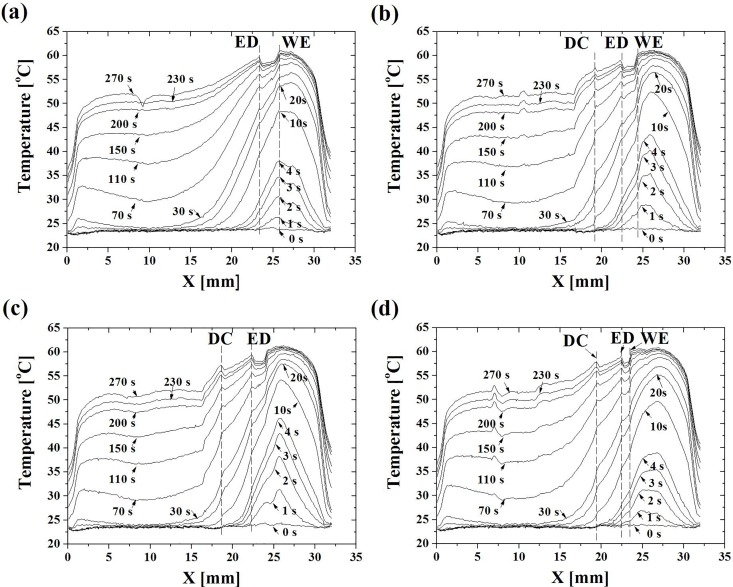
Temperature distributions along four typical buccal lines of the section of a human molar at (a) y_1_ = 16.5 mm, (b) y_2_ = 13.5mm, (c) y_3_ = 11.5 mm and (d) y_4_ = 8.5 mm at each time steps from 0s till 270s.

As shown in Fig 4 temperature changes with time at four lines of y = 16.5, 13.5, 11.5 and 8.5mm in Fig 3; each line is chosen along the direction of heat transfer and across different material regions and with various thicknesses in the enamel layer.

At each line, locations with discontinuous jumps in the temperature are shown as dashed lines which correspond to the places at the water-enamel (WE), enamel-dentine (ED) and dentin- pulpal chamber (DC) interfaces and are shown as dashed lines in Fig 4. As seen, at the beginning of the test, room temperature corresponds to the line at 0s; in the following time steps, the temperature distribution in the water region is quite inhomogeneous; the temperature in Fig 4B and 4C is slightly higher than that in Fig 4A and 4D at a given time step; each curve has its highest temperature at the center of water and decreases along the x-direction. A significant temperature drop can be observed for water-enamel interface at 40°C (10s) and demonstrates the temperature dependence (Fig 4B and 4C). With the temperature approaching the saturation point, i.e. 60°C, the rate of the temperature drop slows down. The temperature drop due to the water-enamel interface can reach about 10°C from 52°C to 42°C at 4s (Fig 4B); while, the temperature of the DEJ is about 37°C and that of the surrounding area is about 54°C (Fig 4C); this is comparable to what would happen by drinking a glass of water for example. This would suggest that one enamel’s role is to provide thermal insulation for the interior structures.

Note that negative thermal resistance only appears above 42°C and becomes significant at 52°C. During this process, it may be associated with collagen denaturation in the DEJ and dentine materials and the effective temperature in the region of pulp close to the dentine layer is about 31.5°C an area rich in both peripheral nerves and blood vessels.

**Fig 5:**
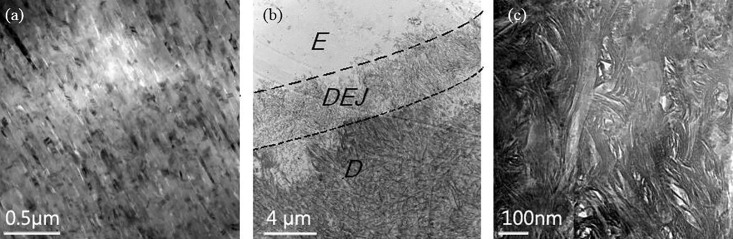
TEM image of human enamel, dentin-enamel junction and dentine. The heat flow is from enamel(E) to dentine(D), (b) is the DEJ image after demineralization. (a):enamel, (b):dentin-enamel junction, (c):dentine

The microstructure details of enamel, dentine and the dentin-enamel junction of human tooth after demineralization by TEM images as shown in Fig 5. We propose that these microstructures play a protective role exploiting the internal material mechanism of both the enamel for sustaining the mechanical stresses, anti-wear and heat resistance and the dentine for the stress and heat dissipation. As is shown Fig 5A, the enamel microstructure is regular composed of 95% mineral (HAP crystals) and limited collagen, while the dentine microstructure has 30% volume of organic matrix (Fig 5C). Across the DEJ interface, the collagen content significantly increases from about zero to about 30% in the dentine material. In addition one can observe an increasing diameter of the collagen fiber from 10nm in the DEJ to about 60-100nm in the dentine layer. When the heat flux transfers from the enamel layer and reaches 42°C in the DEJ, collagen denaturation begins; this leads to a peak value above 42°C at the DEJ as shown in Fig 4. Notice that the peak temperature appears at the dentine and pulp interface in Fig 4, which is due to the difference in the cell and soft tissue enrichment of dentine material compared to the pulp. It is also important to know that the temperature of 42°C coincides with that at which collagen denaturation occurs though it is a temperature at which that living tissue can survive.

## Discussion

Interestingly, the behavior of the temperature hump effects at the DEJ (x~22.5mm) and dentin-pulpal chamber junction (DCJ) (x~18.5mm) only appear above the critical temperature of 42°C while there is no hump effect below 42°C. In other word, the thermal contact resistance of DEJ (*R*_*c*_) is zero below that level. This implies that continuous models could be used for the numerical prediction of the temperature distribution in a low temperature regime without having structural details of the DEJ. Another interesting point for these temperature hump effects at the interfaces of DEJ and DCJ is observed with the negative thermal resistance (*R*_*c*_ < 0) behavior above 42°C. In the low temperature regime, the temperature distributes continuously across the junctions and there are no temperature jumps. This can be explained by the heat accumulation occurring at the junctions, which relates to the physiological function of these junctions. A function of the DEJ is to slow down the heat transfer process in a low temperature regime by building up the heat accumulation; when it reaches 42°C, it is replaced by another mechanism; that is, the junction can sustain that amount of heat flux and then the self-heating behavior at the junction occurs which then results in an increase in temperature.

**Fig 6:**
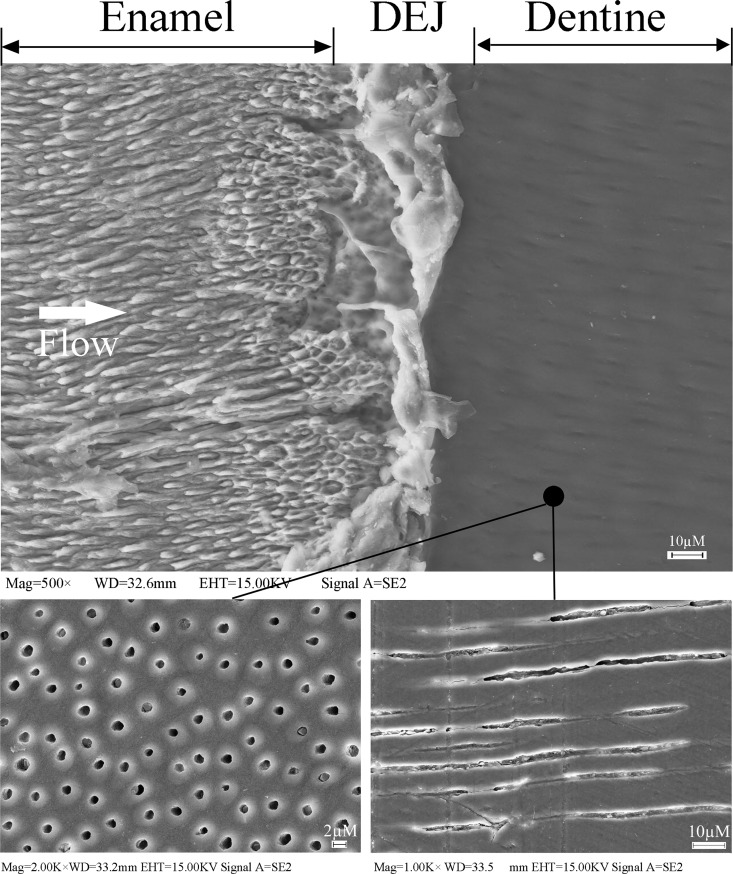
SEM image of human dentine microstructures showing solid dentine material and tubules. The heat flow is perpendicular to the DEJ interface.

The thermal diffusivity *α* of water at 20°C is about 5990 Wm^-1^K^-1^, silver plate is 290 Wm^-1^K^-1^, and that of the insulation of plastic materials is at the level about 0.04 Wm^-1^K^-1^ at room temperature. Therefore, the measured *α* of the enamel and dentine materials are 0.81 and 0.48 Wm^-1^K^-1^, which are good at thermal insulation. This behavior may be explained by the porous microstructures in the dentine layer and the gradient variation of the microstructures from the enamel layer to the dentine layer. The difference in the microstructures for the dentine and enamel layer is shown by the SEM-images in Fig 6. Note that dentin is a mineralized connective tissue with an organic matrix of collagenous proteins. Dentin has microscopic channels, called dentinal tubules, which radiate outward through the dentin from the pulp cavity to the exterior cement or enamel border. The diameter of these tubules range from 2.5 μm near the pulp, to 1.2 μm in the midportion, and 900 nm near the dentin-enamel junction. Previous studies on the thermal behaviors for the dentine materials demonstrated that the microstructure arrangement of the dental layer has a significant effect on overall thermal diffusivity values [24,30]. Also, they may have tiny side-branches and the tubules do not intersect with each other. The three dimensional configuration of the dentinal tubules is complex in particular in the region close to the DEJ boundary, which is believed to play an important role on the thermal behavior. Furthermore, it may also involve the thermal stress due to the difference in the *α*-values of the enamel and dentine materials. Interestingly, Shimizu and Macho[31] proposed that the scalloped architecture within the DEJ may be a mechanism to protect against enamel delamination from the dentine core during mastication. Further the correlation between degree of decussation and scallop magnitude suggests that scallops may have been formed in response to high bite forces.

The thermal insulating capability is measured with thermal conductivity (*k*). Low thermal conductivity is equivalent to high insulating capability (*R*-value). In thermal engineering, other important properties of insulating materials are product density (*ρ*) and specific heat capacity (*c*). Human teeth have layered composite structure of complex inhomogeneous geometry, as shown in Fig 1. The thermal behavior of teeth is mainly a heat conduction process coupled with complicated physiological processes. The thermophysical properties of teeth vary from one layer to another, and are anisotropic even within the same layer, as demonstrated in the present study. Having a better understanding of the thermophysical properties of human teeth could help in choosing better dental restorative materials and avoiding post-operative complications. Studies on teeth heat transfer can also provide a better approach to evaluate thermal treatments. Furthermore, a better understanding of temperature distributions, heat transfer phenomenon and related thermomechanics in teeth will add to the existing knowledge on mechanisms underlying thermal pain, as well as of thermal pain relief.

Combining IR thermography with the monotonic heating scheme to study heat transfer in teeth can not only provide a simple method for non-destructively measuring the thermal properties of individual tooth layers, but also to provide spatially resolved temperature distributions on the tooth surface. Even when large temperature gradients are present at the interfaces of tooth layers, this method is capable of providing reliable measurements of tooth surface temperatures, which the traditional thermocouple-based method fails to do. In addition to tooth thermal properties, the proposed method also provides information about surface heat transfer coefficient, surface adiabatic effectiveness and enamel/dentine junction thermal resistance.

Dental composite resins are types of synthetic resins widely used in dentistry as restorative material or adhesives for the insolubility, aesthetics, dehydration insensibility, easy manipulation and reasonably cost. They are most commonly composed of Bis-GMA and other dimethacrylate monomers which are of the excellent thermal insulation materials with the tailoring thermal conductivities from 0.01 Wm^-1^K^-1^ to 0.05 Wm^-1^K^-1^, when the selection of dimethylglyoxime as additives to achieve certain physical properties, such as flow ability and silica as the filler materials, is considered in most current applications of modern restorative materials and techniques to reliably seal cavity preparations [32]. In these cases, the selection of the restorative materials only considers certain physical properties such as the thermal protection, mechanical strength and material reliability in the oral microenvironments but without the negative thermal resistance. However, a better design can be achieved by formulating unique concentrations of each constituent and exploiting the characteristics of their microstructures. In this point, the thermal behavior of the human DEJ structure in this paper provides the better understanding of in the design.

## Conclusion

In summary, by using infrared thermography, the whole-field temperature distribution over time was determined for a tooth despite its complex geometry and material structures. Due to the different thermal properties of enamel and dentine, we were able to calculate a temperature gradient on the tooth surface. A simple temperature boundary condition was applied using the water circulation system on the outer crown surface of the tooth section starting from room temperature. Changes in temperature data along the direction of the heat transfer were used for deducing thermal diffusivity of the enamel and dentine materials and the results were about 0.81 Wm^-1^K^-1^ and 0.48 W m^-1^K^-1^, respectively. The calculated values are close to the measured temperature drop across the enamel layer of about 10°C observed in the current configuration, which suggests a functional role for the enamel layer. Moreover, the thermal resistance of the DEJ at temperatures above 42°C, a critical temperature below which living tissue can survive, implies that the microstructures of these junctions play an important physiological role.
